# Spike-Driven Neuromorphic Sensing for Energy-Proportional Indoor Air Quality Monitoring in Multi-Zone IoT-Enabled Smart Building Environments

**DOI:** 10.3390/s26133992

**Published:** 2026-06-24

**Authors:** Luigi Carlo M. De Jesus, Aaron Don M. Africa, Ana Antoniette C. Illahi, Reggie C. Gustilo, Stanley Glenn E. Brucal

**Affiliations:** Department of Electronics, Computer, and Electrical Engineering, De La Salle University, 2401 Taft Avenue, Manila 0922, Philippines; luigi_dejesus@dlsu.edu.ph (L.C.M.D.J.); ana.illahi@dlsu.edu.ph (A.A.C.I.); reggie.gustilo@dlsu.edu.ph (R.C.G.); stanley_brucal@dlsu.edu.ph (S.G.E.B.)

**Keywords:** IoT sensor networks, neuromorphic computing, spiking neural networks, leaky integrate and fire, cross-paradigm evaluation, event-driven processing, spike-timing-dependent plasticity, energy-efficient sensing, adaptive Kalman filter, sparse coding

## Abstract

**Highlights:**

**What are the main findings?**

- A spike-driven neuromorphic framework for multi-zone indoor air quality monitoring achieved highly sparse event-driven computation (mean SFR = 10.94%) by detecting and processing only changes in air conditions and ignoring redundant processing, thereby preserving the responsiveness of the environment to changes in air quality across nine zones of the building.
- The presented framework showed a computational cost that was approximately 8.9 times lower than that of LSTM inference (approximately 89% reduction) based on floating-point operation (FLOP) estimation and performance saturated at four neurons per IAQ parameter.

**What are the implications of the main findings?**

- The results show that neuromorphic sensing can be a scalable and energy-proportional alternative to continuous cloud-based IAQ analytics, particularly in distributed smart building environments where bandwidth and power are limited.
- The validated PEI and CES evaluation metrics furnish a transferable framework for comparing event-driven and conventional AI models, which will guide future deployment decisions in smart buildings, edge IoT, and environmental intelligence systems.

**Abstract:**

Indoor Air Quality (IAQ) monitoring, especially in multi-zone smart buildings, is typically limited by the high computational and energy requirements of continuous sensor processing, which makes event-driven methods desirable for efficiency. Energy proportionality, in this context, refers to a system whose computational cost scales with the significance of detected environmental changes rather than with the fixed sampling rate. This paper presents a spike-driven neuromorphic sensing framework for decentralized IAQ monitoring that combines adaptive Kalman filter preprocessing, dynamic threshold-based asynchronous spike encoding, and a Leaky Integrate-and-Fire neural network with Spike-Timing-Dependent Plasticity (STDP) learning. Multiple-parameter IAQ data including PM_1_, PM_2.5_, PM_10_, CO_2_, CO, TVOCs, and O_3_ were sampled from nine functionally differing zones of an educational building in Metro Manila, Philippines. The neuromorphic model yielded a mean Sparse Firing Ratio of 10.94%, a Mean Response Time of 10.62 timesteps, and an energy efficiency proxy score of 9.28. Neuron population scaling and parameter robustness analyses revealed that the four neurons per parameter were enough to saturate the performance, and FLOP-based estimation indicated an 8.9-fold computational reduction (approximately 89% fewer FLOPs) compared to LSTM inference. In addition, the revised Performance Efficiency Index and composite efficiency score corroborated the stable and energy-proportional nature of behavior in all zones. These results illustrate that spike-based neuromorphic computation is an energy-efficient and scalable way for decentralized smart-building IAQ monitoring, though hardware-level validation on dedicated neuromorphic processors remains necessary for absolute power saving verification. Multi-seed validation (five seeds) with expanded baselines including GRU, Temporal CNN, XGBoost, and Logistic Regression confirmed the robustness and repeatability of reported metrics.

## 1. Introduction

The wide deployment of Internet-of-Things (IoT) sensor networks in smart buildings has made it possible to monitor indoor environments constantly and in great detail. However, traditional sensor systems are still facing the limitations of excessive data transmission, central computing bottlenecks, and high energy consumption compared to the amount of useful information they provide [1,2,3]. Indoor air quality (IAQ) is one of the most important factors affecting the health of building occupants, their cognitive abilities, and their overall productivity. Many studies have found that long-term exposure to high levels of CO_2_, fine particulate matter (PM_2.5_ and PM_10_), and volatile organic compounds (VOCs) can lead to several serious health issues, such as respiratory, cardiovascular, and neurocognitive problems [4,5]. Multi-sensor IoT systems have greatly enhanced both the spatial and temporal coverage of IAQ measurement [6,7]. Still, the most common approach—continuous sampling and cloud-based processing—brings about delays, limits in the bandwidth and problems with scaling up, which are particularly pronounced in large multi-zone buildings [8,9].

Computational intelligence developments for building systems that include digital twin modeling [10], fog computing architectures [8], and deep learning-based prediction [11,12] have partially mitigated these limitations. That said, these techniques mostly depend on massive and uninterrupted data streams that must be processed through traditional von Neumann architectures, which leads to computational loads that increase linearly with the number of sensors and the frequency with which data are sampled [13,14]. The sound principles of biological neural systems, which use little activation and are event-driven, have inspired neuromorphic computing that represents a radically different concept of sensor data processing [15,16,17].

Spiking neural networks (SNNs) are capable of information exchange solely via individual spike events that are triggered by a significant stimulus. This allows for avoiding computational overload related to processing of time-wise redundant data [18]. Precision agriculture [19], acoustic classification [20], urban environmental monitoring [21], and edge IoT deployment [22,23] are just some of the examples where this event-driven methodology has shown considerable benefits. In addition, spiking computation is inherently energy proportional in the sense that the cost of processing depends on the significance of an event, not on its frequency of sampling. This is the feature that makes it well-suited for distributed sensor networks in buildings, where there are constraints on energy and bandwidth [15]. Even though this is the case, the use of neuromorphic processing for monitoring indoor air quality is still far from being realized. Present-day SNNs have been applied mainly to single-modality sensors or to highly controlled environments [17,22], which means that a multi-parameter, multi-zone IAQ analysis for real-world building scenarios is a major area of research that has not been tackled yet. Moreover, since there are no standardized evaluation frameworks for event-driven versus continuous processing models that recognize their fundamentally different computational objectives, it is difficult to carry out meaningful cross-paradigm comparisons.

Through this paper, the authors address these gaps via three contributions: (1) designing and implementing a spike-based neuromorphic processing framework for multi-parameter IAQ data across nine functionally diverse building zones, incorporating neuron population scaling analysis and multi-seed robustness validation with expanded baselines (GRU, Temporal CNN, XGBoost, and Logistic Regression); (2) coupling adaptive Kalman filter preprocessing with dynamic threshold-based spike encoding, thus generating an extremely robust signal conditioning chain; and (3) formulating revised cross-paradigm evaluation metrics—Performance Efficiency Index (PEI) and composite efficiency score (CES)—which facilitate rigorous comparison between event-driven neuromorphic and continuous machine learning paradigms.

## 2. Related Works

### 2.1. IoT-Based Indoor Air Quality Monitoring Systems

Multi-sensor data fusion is now a basic plan for IAQ prediction improvement in highly changing building situations [1,6]. Floris et al. [6] proved with experiments that using different IoT sensors helps a lot in predicting the number of people and in sorting the environmental situations in smart buildings. Ha et al. [24] showed that adaptive Kalman filtering, by updating its states, can lead to very robust pollutant estimations even when the sensor characteristics change nonlinearly. Lopes et al. [25] gave a detailed review of how to associate low-cost IAQ sensors with IoT technology and calibration techniques in the field. Architectures for distributed monitoring have been further developed via LoRaWAN-based networks for controlling ventilation over a large scale [26] and ontology-driven data representations for cross-domain decision-making [27]. Dong et al. [12] tackled artificial neural network models for IAQ prediction in schools and sensor noise was identified as one of the main problems. The SEMAR-VAaMSN framework [28] got to sub-millisecond data abstraction latency by means of microservice-based architectures. Despite all this, the main drawback of continuous data processing is still there [8,9].

### 2.2. Neuromorphic Computing for Environmental Intelligence

Neuromorphic computing architectures have shown major potential in environmental sensing, at least in theory. Lu and Xiao [19] observed up to 47% reductions in energy consumption in smart greenhouse systems using spiking neural network processing [17]. Xue et al. developed EdgeMap, a toolchain that maps spiking neural networks to neuromorphic hardware for edge-based computing tasks. Additionally, Morshed et al. [18] found that stochastic spiking models perform better than deterministic analog networks in adapting to changing conditions and resisting noise. Yan and Qiu [15] argue that neuromorphic designs are vital for next-generation sensing with low power demands. Park et al. [22] added SNN-to-hardware mapping strategies that reduce network-on-chip latency, improving IoT sensor efficiency. Sungheetha et al. [16] realized notable energy savings with adaptive stream processing in greenhouse monitoring systems. It seems difficult to ignore the gap in applying these methods to multi-zone indoor spaces with diverse pollutant levels.

### 2.3. SNN Applications in the IAQ Monitoring Domain

Despite the growing body of work on SNNs for environmental sensing, the application of spiking neural networks specifically to indoor air quality monitoring remains largely unexplored. Existing SNN research has focused on agricultural monitoring [16,19], acoustic event detection [20], and olfactory sensing [21], where the event-driven nature of spikes aligns well with episodic environmental changes. However, indoor air quality monitoring presents distinct challenges: it requires simultaneous processing of multiple correlated pollutant parameters (PM, CO_2_, VOCs, and O_3_), the monitored environment exhibits heterogeneous spatial and temporal variation across different functional zones, and the definition of actionable events depends on regulatory thresholds rather than naturally discrete stimuli. None of the existing SNN works address the combined challenge of multi-parameter, multi-zone IAQ processing, nor do they provide evaluation frameworks that allow for a principled comparison between event-driven and continuous processing paradigms.

### 2.4. Research Gaps and Contributions

Existing publications highlight a considerable challenge at the intersection of neuromorphic computing and indoor air quality (IAQ) monitoring. Despite their ability to provide high-resolution sensing [1,6,25], IoT-based IAQ systems are still confined to continuous processing methods. On the other hand, neuromorphic computing has been shown to be an energy-efficient event-driven processing method in agricultural [16,19] and acoustic [20] domains, but it has not been applied to multi-parameter building environments. More importantly, none of the existing works deliver: (a) a neuromorphic framework capable of processing seven simultaneous IAQ parameters across nine different building zones, along with neuron scaling analysis; (b) a combination of adaptive signal conditioning and spike encoding for environmental sensor data; or (c) composite evaluation metrics that allow for a principled cross-paradigm comparison. To the best of the authors’ knowledge, no prior work has simultaneously addressed all three issues. This paper addresses all three.

## 3. Materials and Methods

### 3.1. Framework Architecture

**Figure 1:**
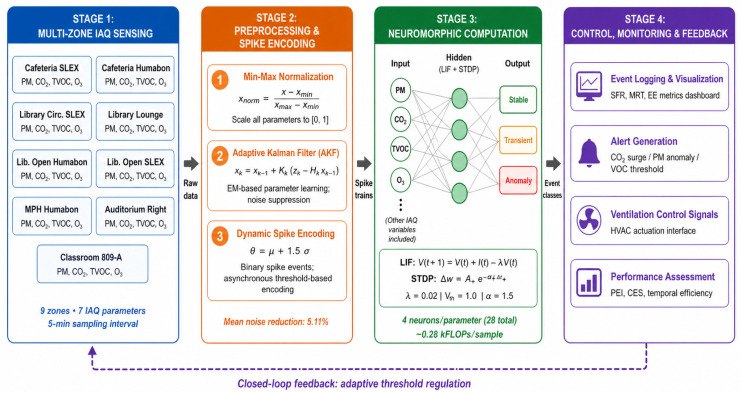
Hierarchical architecture of the proposed spike-driven neuromorphic IAQ monitoring framework.

The proposed framework employs a hierarchical four-tier architecture for event-driven IAQ processing, as illustrated in Figure 1. The Sensor Network Stage acquires environmental parameters through distributed IoT nodes deployed across nine functionally distinct building zones. These raw measurements are then passed to the Preprocessing Stage, which applies adaptive Kalman filtering for noise attenuation, min–max normalization, and dynamic threshold-based spike encoding to convert continuous sensor signals into asynchronous binary events. The resulting spike sequences feed into the Neuromorphic Computation Stage, where Leaky Integrate-and-Fire neurons with Spike-Timing-Dependent Plasticity learning perform temporal integration and event classification. Finally, the Control and Monitoring Stage decodes the classified spike outputs and relays control signals to ventilation management interfaces, with a feedback path enabling closed-loop adaptive regulation.

### 3.2. Data Acquisition, Sensor Specifications, and Preprocessing

Environmental data were collected from nine zones within an educational building complex in Metro Manila, Philippines. These zones included classrooms, libraries, cafeterias in the South Luzon Expressway (SLEX) and Humabon wings, an auditorium, and Multipurpose Hall 1 (MPH1). Each zone was fitted with IoT sensor nodes that recorded PM_1_, PM_2.5_, PM_10_, equivalent CO_2_/eCO_2_, CO/VOC-related multichannel gas response, TVOCs, O_3_, dry-bulb temperature, and relative humidity.

The deployed sensor modules were the Grove SEN5X All-in-One Environmental Sensor (Seeed Studio, Shenzhen, China) for PM_1_ (±5 µg/m^3^ and ±5% of measured value from 0–100 µg/m^3^; ±10% from 100–1000 µg/m^3^), PM_2.5_ (±5 µg/m^3^ and ±5% of measured value from 0–100 µg/m^3^; ±10% from 100–1000 µg/m^3^) and PM_10_ (±25 µg/m^3^ from 0–100 µg/m^3^; ±25% from 100–1000 µg/m^3^); dry-bulb temperature (±0.45 °C); and relative humidity (±4.5% RH); Grove VOC and eCO_2_ Gas Sensor SGP30 (Seeed Studio, Shenzhen, China) for CO_2_, retained in this study as the equivalent CO_2_/eCO_2_ channel (400–60,000 ppm output range), and total volatile organic compounds (TVOCs) (0–60,000 ppb output range); Grove Multichannel Gas Sensor V2 (Seeed Studio, Shenzhen, China) for CO/VOC-related multichannel gas response (qualitative multichannel gas response); and the MQ131 Ozone Gas Sensor Module (Zhengzhou Winsen Electronics Technology Co., Ltd, Zhengzhou, China) for O_3_ (10–1000 ppb detection range for the low-concentration variant). Particulate matter was detected using the laser-scattering method, while gas-channel readings were obtained from low-cost MOS/MEMS semiconductor gas sensors. The CO_2_ channel is retained as “CO_2_” in the tables and results for consistency with the original dataset labels, although it refers to the equivalent CO_2_/eCO_2_ output of the SGP30 sensor. All sensors were factory calibrated; there was no field recalibration or cross-sensitivity correction applied, which is a limitation that is discussed in Section 5.7. Sensor nodes sent data every five minutes along with the synchronized timestamp. Missing data were filled through a linear interpolation method; however, data gaps longer than ten minutes were discarded.

The IoT-based sensor network consisted of Grove sensors interfaced with Arduino Uno (Arduino SA, Chiasso, Switzerland) and Raspberry Pi (Raspberry Pi Ltd., Cambridge, UK). Arduino Uno served as the analog-to-digital converter (ADC) for the MQ131 ozone sensor and the integrator hub for other Grove sensors. Serial communication between Arduino and Raspberry Pi was performed only when data were being transmitted. A Python script scheduled on crontab acquired data every five minutes, parsing the sensor readings and validating only those records where exceptions or missing values were found. Real-time and historic data were stored in a MariaDB database on Raspberry Pi, with the database schema designed for scalability and efficient querying [9,14,15]. Table 1 shows the brief descriptive statistics of the IAQ dataset. The validated operational ranges were based on the deployed sensor modules: CO_2_ between 400 and 60,000 ppm as equivalent CO_2_/eCO_2_ from the SGP30 sensor, CO/VOC-related multichannel gas response from the Grove Multichannel Gas Sensor V2, relative humidity from 0 to 100% RH, temperature from −40 to 125 °C, O_3_ from the MQ131 ozone sensor module, PM_1_/PM_2.5_/PM_10_ from 0 to 1000 µg/m^3^ using the SEN5X laser-scattering particulate sensor, and TVOCs from 0 to 60,000 ppb using the SGP30 sensor. Each sensor node recorded nine parameters at five-minute intervals, producing approximately 720 bytes/hour per node (60 bytes/record × 12 records/hour). Across nine nodes, the total data throughput was approximately 155.5 KB/day (~1.8 bytes/second), representing negligible bandwidth requirements.

Preprocessing was divided into three consecutive steps. Initially, all features were normalized to the range [0, 1] using min–max scaling (Equation (1)): This normalization formula follows standard machine learning practice and is not a novel contribution of this work.

x_norm_ = (x_i_ − x_min_)/(x_max_ − x_min_), (1)

After this, the stochastic sensor noise was drastically attenuated by an adaptive Kalman filter (AKF), which kept at least some significant changes of the signal over time. The refresh of the filter throughout the processing was based on a classical recursive Kalman update (Equation (2)):

x_k_ = x_k−1_ + K_k_(z_k_ − H_k_ · x_k−1_), (2)

where K_k_ stands for the Kalman gain, z_k_ is the incoming measurement, and H_k_ is the measurement matrix. The filter was started with the very first measurement, and its transition and measurement parameters were obtained through expectation–maximization, which allowed for adaptive noise estimation without manually tuning individual sensors. This standard Kalman filter formulation follows from the seminal work of Kalman (1960) and has been widely adopted for sensor fusion [24,29]. Even though the original research plan classified this element as fractional-order, the implemented filter is a standard-order Kalman filter with adaptive parameterization; this change is marked here explicitly for the sake of methodological clarity. The adaptive parameterization brought about consistent noise reduction over different types of sensors. At last, denoised signals were translated into binary spike sequences by dynamic thresholding (Equation (3)):

θ = μ + ασ,(3)

where μ and σ stand for the mean and standard deviation within the local window, and α = 1. 5 stands for a sensitivity constantly optimized empirically. Then, binary spike events were generated whenever the threshold was exceeded, which led to asynchronous spike trains.

### 3.3. Neuromorphic Computation Model

**Figure 2:**
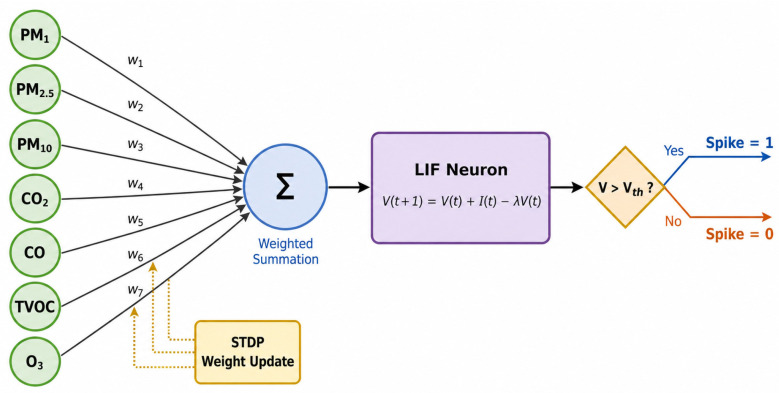
LIF neuron architecture in MISO (Multiple Input Single Output) configuration.

The Leaky Integrate-and-Fire (LIF) neuron model serves as the computational core. The dynamic membrane state V(t) obeys Equation (4):

V(t + 1) = V(t) + I(t) − λV(t),(4)

where I(t) stands for the weighted input current, while λ = 0.02 represents the membrane leakage constant. Once the threshold, V_th_, is exceeded, the neuron discharges a spike and resets itself. The neuron architecture follows a Multiple Input Single Output (MISO) configuration: each LIF neuron receives spike-encoded inputs from seven IAQ parameters, integrates them through weighted summation, applies the leaky integration function, and produces a binary spike output when the membrane potential exceeds V_th_. Synaptic modification is carried out through the STDP method (Equation (5)): The MISO neuron architecture is illustrated in Figure 2.

Δw = A^+^ exp(−Δt/τ^+^) if t_pre_ < t_post_; −A^−^ exp(−Δt/τ^−^) if t_pre_ > t_post_, (5)

This formula increases the strength of temporally linked connections and decreases it for those in which the activities are not correlated. Thus, the network can learn to detect changes or respond differently to the input without any form of supervision training [15,18]. The NN consists of an input layer (seven indoor air quality (IAQ) channels), a hidden layer (LIF-STDP for temporal integration) and an output layer (environmental states classified based on spike density estimation in a 30-sample sliding window). This standard STDP formulation follows from Bi and Poo (1998) [30], where A^+^ = 0.01, A^−^ = 0.012, and τ^+^ = τ^−^ = 20 ms.

### 3.4. Neuron Population Scaling and Robustness Analysis

In order to determine how sensitive the framework is to changes in architectural parameters, two additional experiments were conducted, namely neuron population scaling and parameter robustness testing. Regarding neuron population scaling, the number of neurons for IAQ parameters was changed (1, 2, 4, 8, and 16), chosen as powers-of-two progression to systematically identify the saturation point, but the input spike streams were kept the same in each case. Moreover, each neuron received a little threshold jitter (σ = 0.05) and leakage jitter (σ = 0.005) to mimic hardware variability. Then, parameter robustness testing involved three different LIF configurations, i.e., τ = 15, V_th_ = 0.9, and λ = 0.015; τ = 20, V_th_ = 1. 0, and λ = 0.020; and τ = 25, V_th_ = 1. 1, and λ = 0.025, with each configuration having eight neurons. The idea behind these experiments is to figure out if the framework can be practically and reliably used on different hardware configurations that would show the variability in manufacturing.

### 3.5. Evaluation Metrics

Three main indicators were used for performance evaluation. Sparse Firing Ratio (SFR) represents the proportion of active spike events relative to the total available spike opportunities (Equation (6)):

SFR = (E_active_/E_total_) × 100%, (6)

Mean Response Time (MRT) was defined as the time interval between threshold violation and spike generation. Energy efficiency (EE), computed as the reciprocal of average spike density, served as a simulation-based proxy for computational energy cost rather than a direct hardware-level power measurement [15,22]. To compare event-driven neuromorphic processing with continuous baseline models, the Performance Efficiency Index (PEI) and composite efficiency score (CES) were introduced, as expressed in Equations (7)–(10). In the revised formulation, balanced accuracy was used as the common performance numerator for both neuromorphic and baseline models. For continuous baseline models, the energy cost was normalized to 1.0 to represent continuous computation, whereas for the neuromorphic model, the activity cost was represented by the Sparse Firing Ratio (SFR). This formulation establishes a unified performance dimension while preserving the different computational cost structures of event-driven and continuous-processing paradigms. Equations (6)–(10) are proposed by the authors of this study as part of the evaluation framework.

PEI_baseline_ = Balanced Accuracy/Energy Cost, (7)

PEI_neuromorphic_ = Balanced/Activity Cost, (8)

TE = 1/Response Time,(9)

CES = PEI × TE,(10)

where Energy Cost=1.0 for continuous baseline models and Activity Cost=SFR for the neuromorphic model.

All the simulations have been coded in Python 3.12 with GPU acceleration (Google Colab, NVIDIA T4 GPU, 16 GB VRAM) using NumPy 2.0.2, Pandas, scikit-learn, TensorFlow/Keras (for LSTM and GRU baselines), and XGBoost. The neuromorphic LIF-STDP model was computed on CPU as it is inherently lightweight. LSTM (32 units, dropout 0.2, 10 epochs; selected per standard IAQ literature practice [5,12]) and linear regression baselines were evaluated with an 80/20 temporal split without shuffling. The authors recognize that these baseline architectures are fairly simple; stronger baseline architectures, such as GRU, Temporal CNN, or Transformer-lite models, could achieve higher levels of accuracy, and the comparisons reported here should be interpreted keeping this in mind. In the initial study, simple LSTM and linear regression were chosen to establish a first-order comparison of event-driven vs. continuous processing paradigms, prioritizing interpretability of the computational cost comparison. Recognizing this limitation, expanded baselines (GRU, Temporal CNN, XGBoost, and Logistic Regression) were subsequently evaluated in the multi-seed validation (Section 4.7) to provide a more rigorous benchmark. 

To incorporate the energy comparison beyond the EE proxy, the computational complexity was estimated by the number of floating-point operations (FLOPs) per inference sample. The LSTM baseline (32 units, 7 input features) uses about 2. 50 kFLOPs per sample (it is a combination of input gate, forget gate, cell state, and output gate computations). The neuromorphic LIF model (4 neurons × 7 parameters = 28 neurons, with each neuron doing one addition, one multiply, and one comparison per timestep) comes to approximately 0.28 kFLOPs per sample. This results in an estimated computational reduction factor of 8.9. Nevertheless, this FLOP-based estimate is a simulation proxy and does not take into consideration memory access patterns, hardware-specific energy per operation, or actual power draw on neuromorphic versus von Neumann hardware. Hardware-level validation on dedicated neuromorphic processors is still needed to ascertain absolute energy savings.

### 3.6. Axiomatic Justification of PEI and CES

PEI and CES are two metrics that were formulated in this study to be used for cross-paradigm comparison of event-driven and continuous processing models. As the next step of confirming their validity, the following analysis demonstrates their defining properties and also does some boundary analysis.

- Property 1 (Non-negativity): Because SFR, accuracy, EE, and response time are all non-negative by their very nature, PEI and CES have to be greater than or equal to zero for any models and zones.
- Property 2 (Monotonicity): PEI is directly proportional to the main performance metric of the model (balanced accuracy for both paradigms) and inversely proportional to energy consumption. In the revised formulation, both neuromorphic and baseline PEI share balanced accuracy as the common numerator, ensuring that an improvement in detection performance increases PEI for any model. CES benefits even more from temporal responsiveness (lower MRT). These monotonic connections guarantee that an increase in any individual dimension results in a similar increase in CES, thus paradoxical rankings are eliminated.
- Property 3 (Dimensional consistency): PEI takes the model’s performance and divides it by the energy cost so that the result is a pure number representing “performance per unit energy.“ CES incorporates temporal efficiency by multiplying PEI by the inverse response time so that a 3D comparison of trade-offs is possible: between the detection ability, the energy used, and the time it takes that characterizes a sensor network deployment.
- Property 4 (Boundary behavior): If SFR decreases while detection performance is preserved, $PEI_{neuromorphic}$ increases because fewer spike events are required per unit of balanced accuracy. However, if SFR approaches zero because the model fails to generate necessary event responses, the model becomes operationally invalid and CES is penalized through degraded balanced accuracy and an undefined or delayed response time. Thus, the metric rewards sparse but functional firing, not silent non-response. If the response time approaches infinity, TE approaches zero and CES also approaches zero, thereby penalizing unresponsive systems.

- Property 5 (Cross-paradigm comparability): In the revised formulation, both neuromorphic and baseline PEI use balanced accuracy as the common performance numerator, resolving the dimensional incommensurability of the original formulation. The denominators differ to reflect paradigm-specific cost structures: Activity Cost (SFR) for neuromorphic and Energy Cost (1.0) for continuous baselines. This enables direct numerical comparison across paradigms.

## 4. Results

### 4.1. Adaptive Kalman Filter Performance

Table 2 shows the noise variance reduction through AKF preprocessing in all nine zones under surveillance. The mean noise reduction was 5.11% by the filter, and the range was 1.32% (Multipurpose Hall 1 (MPH1) Humabon Side) to 19.37% (809 A Classroom). The 809 A Classroom’s large increase is a reflection of the substantial temporal noise in CO_2_ and VOC signals caused by occupancy changing at intervals. Areas with stable air movement had little or no reduction, which proves that AKF is a method that can be used to remove stochastic noise only and at the same time keep the changes in the environment that are meaningful.

### 4.2. STDP Weight Dynamics and Spike Raster Analysis

**Figure 3:**
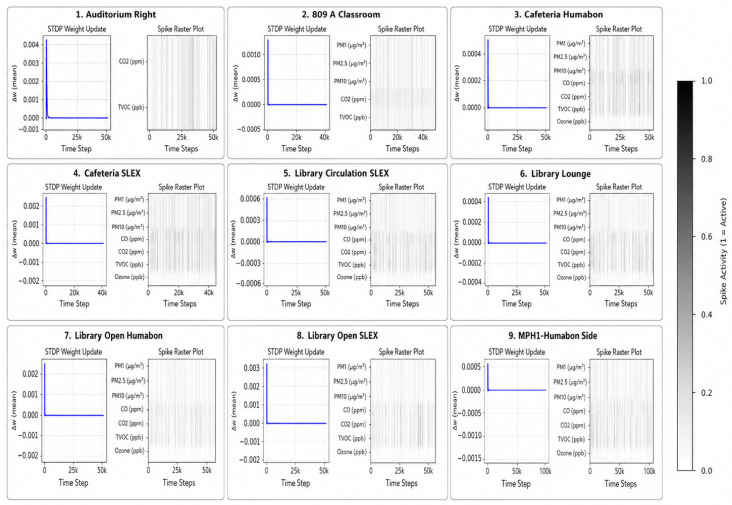
Combined STDP weight update curves (**left**) and spike raster distributions (**right**) for all nine monitored zones.

Figure 3 presents the STDP weight-update behavior and corresponding spike raster distributions for the nine monitored zones. In each zone panel, the left subpanel shows the mean STDP weight change over time, while the right subpanel shows the spike raster activity across the monitored IAQ parameters. The cafeteria zones, particularly Cafeteria SLEX and Cafeteria Humabon, exhibit more visible spike activity across particulate and gaseous parameters, suggesting stronger synaptic adaptation to rapid IAQ fluctuations associated with food-service activity, CO_2_ variation, and TVOC changes. The library zones generally show more stable STDP trajectories and relatively sparse spike activity, indicating lower pollutant variability and more consistent environmental conditions. The auditorium and multipurpose hall show near-static STDP responses with low-density firing patterns, suggesting limited spike activation during the observed period. The 809 A Classroom shows a comparatively balanced response, with moderate spike activity across selected IAQ variables, indicating adaptive plasticity under variable occupancy and airflow conditions. Overall, the figure shows that the neuromorphic model dynamically adjusts synaptic activity and firing density according to zone-specific environmental variability, supporting selective responsiveness rather than continuous high-rate computation.

### 4.3. Neuromorphic Model Performance Across Zones

Table 3 consolidates the neuromorphic model results by the zone using the per-zone LIF-STDP simulation data (given by Equations (4) and (5)). The average SFR of 10.94% is in good agreement with the selectivity of sparse activation neurons that did not fire under normal conditions, and firing was observed only when statistically significant deviations occurred. The negative correlation between SFR and EE is indicative of the energy-proportional nature of computation: lower event-frequency zones (Auditorium Right, SFR = 9.72%) attained higher energy efficiency (EE = 10.29), whereas high-activity zones (Cafeteria Humabon, SFR = 12.92%) experienced lower EE (7.74) but environmental responsiveness at a higher level.

### 4.4. Neuron Population Scaling Analysis

Table 4 explores whether adding more LIF neurons per IAQ parameter helps the neuromorphic model perform better. Only one neuron per parameter gave an SFR of 5.52% with an MRT of 22.23 timesteps. Having two neurons resulted in an increase in SFR to 7.09% and a decrease in MRT to just 17.36 timesteps. For four neurons per parameter the model saturated: SFR remained at 11.07%, MRT settled at 11.16 timesteps, and EE at 9.03. Changes to 8 and 16 neurons brought only slightly different metrics, showing that the spike aggregation mechanism (logical OR across the neuron population) reaches functional convergence already at four neurons. In addition, this saturation phenomenon is of great practical significance: it clearly shows that the framework can operate with minimal and maximal event detection capabilities without being dependent on large neuron populations, thus substantially reducing the hardware resource requirements when it comes to edge deployment [22,23].

### 4.5. Parameter Robustness Analysis

Table 5 shows how robust the neuromorphic model is with three different sets of LIF parameters. The first two parameter sets (τ = 15, V_th_ = 0.9 and τ = 20, V_th_ = 1.0) resulted in exactly the same output (SFR = 11.07%, MRT = 11.16, and EE = 9.03), which indicates that the method is not affected by small changes in the values of the threshold and the leakage. The last parameter set (τ = 25, V_th_ = 1.1, and λ = 0.025) increased the firing threshold, which caused SFR to go down (7.38%), MRT to go up (16.33 timesteps), and EE to go up (13.54). The normal compromised higher thresholds that produce sparser but slower firing along with a more efficient usage of energy confirms that the method is capable of delivering predictable, tunable behavior over ranges of parameters that can be exploited for optimization between responsiveness and energy saving for a certain type of deployment.

### 4.6. Comparative Analysis with Conventional Baselines

Table 6 shows the comparison of different paradigms in terms of operations. The neuromorphic SFR and traditional accuracy metrics target different aspects at their core: SFR measures the degree of event-driven sparsity, whereas LSTM and regression accuracy give indications of the classification prediction performance. The neuromorphic setup scored an average EE of 9.28, which is an approximately 89% less computational cost (a factor of 8.9 reduction) than the continuously running baselines (the contrasting method assumes unit energy consumption). Although LSTM was more accurate in very stable conditions (e.g., Auditorium Right, 93.70%), it was less effective in zones with high variability (Library Open Humabon, 47.35%). The neuromorphic model showed that its performance changes were as equal as possible in all the zones.

### 4.7. Expanded Baseline Comparison and Multi-Seed Validation

**Figure 4:**
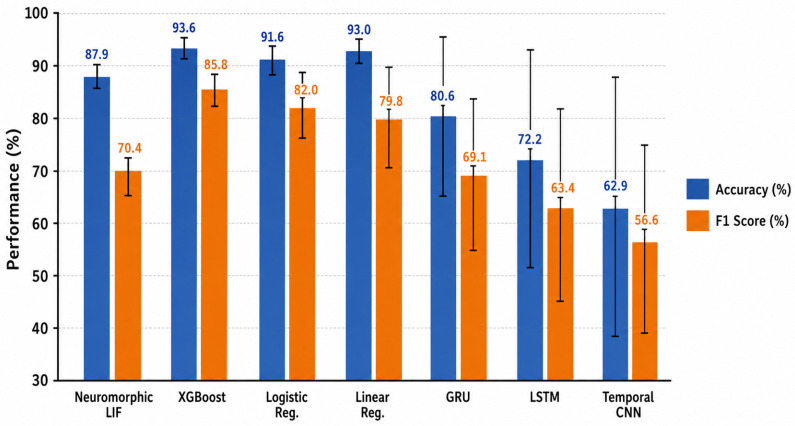
Expanded baseline comparison showing accuracy and F1 score (mean ± SD) across seven models, five random seeds, and nine monitored zones (315 total runs). Error bars represent standard deviation.

To address the limitation of using only simple LSTM and regression baselines, expanded benchmarking was conducted with seven models across five random seeds (7, 21, 42, 84, and 168) and nine rooms, yielding 315 total experimental runs. The following summarizes the aggregated results. XGBoost achieved the highest classification accuracy (93.60 ± 2.12%), followed by linear regression (93.03 ± 2.70%) and Logistic Regression (91.58 ± 3.55%). The neuromorphic LIF model achieved 87.88 ± 2.45% accuracy with markedly lower computational cost (SFR = 7.32 ± 2.13%, MRT = 5.13 ± 1.67 steps, and EE = 14.96 ± 4.94). Deep learning models exhibited high variance, LSTM at 72.20 ± 20.81% and Temporal CNN at 62.89 ± 24.42%, suggesting instability on this dataset size. In terms of F1 score, XGBoost led with 85.77 ± 4.30%, followed by Logistic Regression at 82.00 ± 6.33%, while the neuromorphic model achieved 70.43 ± 5.36%. Figure 4 illustrates the accuracy and F1 score comparison across all models.

### 4.8. Statistical Validation

**Figure 5:**
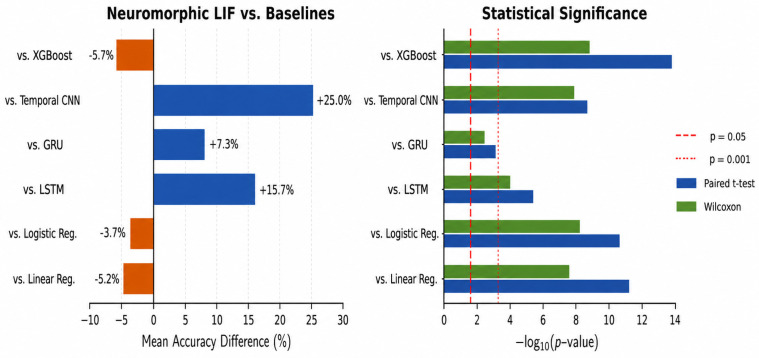
Statistical comparison of neuromorphic LIF versus baseline models. (**Left**) Mean accuracy difference (positive = neuromorphic advantage). (**Right**) Statistical significance (−log_10_ *p*-value); all comparisons exceed the *p* = 0.05 threshold.

Paired *t*-tests and Wilcoxon signed-rank tests were conducted across the 45 paired room-seed comparisons (9 rooms × 5 seeds) for each model pair. All comparisons were statistically significant (*p* < 0.05). The neuromorphic LIF model significantly outperformed LSTM (mean diff. = +15.68%, *p* = 5.45 × 10^−6^), GRU (+7.32%, *p* = 1.12 × 10^−3^), and Temporal CNN (+24.99%, *p* = 1.36 × 10^−8^). Conversely, XGBoost (−5.71%, *p* = 2.23 × 10^−14^), linear regression (−5.15%, *p* = 4.70 × 10^−12^), and Logistic Regression (−3.70%, *p* = 2.49 × 10^−11^) significantly outperformed the neuromorphic model in accuracy. These accuracy differences must be interpreted in conjunction with the 8.9-fold computational cost reduction of the neuromorphic approach. Figure 5 visualizes the mean accuracy differences and significance levels for all pairwise comparisons.

### 4.9. Composite Efficiency Analysis

**Figure 6:**
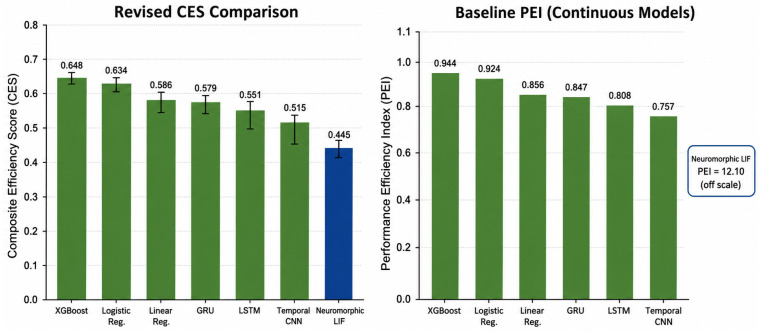
Composite efficiency score (CES) and Performance Efficiency Index (PEI) comparison across models. (**Left**) CES values showing XGBoost and Logistic Regression achieving highest composite scores; neuromorphic LIF shows lower CES due to temporal efficiency penalty but achieves PEI = 12.10 (off-scale, shown in annotation). (**Right**) Baseline PEI values for continuous models.

Table 7 displays the PEI and CES metrics. Using the revised formulation with balanced accuracy for both paradigms (Equations (7)–(10)), the model-level results across five seeds and nine rooms are as follows: Neuromorphic LIF achieved a revised mean PEI of 12.10 ± 3.39 and CES of 0.445 ± 0.040 (reflecting energy-proportional event-driven processing). XGBoost achieved the highest CES at 0.648 ± 0.013 with PEI of 0.944, followed by Logistic Regression (CES = 0.634 ± 0.023 and PEI = 0.924). The neuromorphic model’s lower CES reflects the temporal efficiency penalty (TE = 0.214) inherent to event-driven processing; however, its PEI of 12.10 is approximately 13× higher than continuous baselines, demonstrating superior performance per unit of computational activity. Figure 6 provides a visual comparison of the revised CES and PEI values across all seven models.

Table 7 presents the revised PEI and CES values computed using balanced accuracy as the common performance numerator for all models. Under this formulation, the neuromorphic LIF model achieved the highest PEI value of 12.10 ± 3.39, indicating superior performance per unit computational activity. However, its CES value of 0.445 ± 0.040 was lower than that of XGBoost and Logistic Regression because CES also incorporates temporal efficiency. XGBoost achieved the highest CES value of 0.648, followed by Logistic Regression with 0.634, indicating that these continuous baselines provided stronger overall accuracy–response trade-offs. Therefore, the revised PEI/CES results show that the neuromorphic model is not superior in raw predictive performance, but it offers a stronger energy-proportional advantage through sparse event-driven computation.

### 4.10. Sensitivity Analysis of CES

In order to assess how stable the CES metric is to changes in constituent weighting, a sensitivity analysis was carried out wherein a parametric exponent, α, was embedded in a generalized CES formulation: CES’ = PEI^α^ × TE^(1−α)^;, where α ∈ [0.3, 0.7] adjusts the relative importance given to energy-normalized performance (PEI) versus temporal responsiveness (TE). The original CES (Equations (7)–(10)) matches with α = 0.5 (equal weighting). Table 8 shows the Spearman rank correlation coefficients between the neuromorphic CES zone rankings at α = 0.5 and those obtained at four different α values.

Spearman rank correlations surpassed 0.96 across all tested α values, indicating consistent zone rankings under notable PEI and TE weighting variations. At moderate settings (α = 0.4 and 0.6), rankings remained unchanged, with ρ equaling 0.998. At extreme values (α = 0.3 and 0.7), only one or two adjacent swaps occurred among zones with similar CES scores, such as Library Lounge and Library Open Humabon. The coefficient of variation for neuromorphic CES stood at 0.315, slightly above LSTM’s 0.181 and regression’s 0.186. Now, this greater variability points out distinct separation between high-activity areas, like cafeterias and low-activity zones, such as auditoriums, a pattern maintained regardless of α configuration. The stability of relative rankings across parameter ranges displays that the CES model delivers consistent discrimination without dependency on specific weighting choices.

## 5. Discussion

### 5.1. Energy-Proportional Sparse Activation

The average spike firing rate of 10.94% indicates precise event selection, suppressing nearly 89% of transient sensor signals deemed non-important. This activation pattern mirrors biological systems’ efficiency in resource allocation [15,16,17], diverging from traditional methods that treat every input as potentially meaningful. A computational load reduction of about 8. 9-fold is consistent with prior findings by Lu and Xiao [19] in agricultural neuromorphic contexts, now extended to multi-parameter indoor air quality monitoring. The inverse relationship between SFR and the EE proxy confirms energy-proportional behavior: low-activity zones, such as Auditorium Right, showed lower SFR and higher EE, whereas dynamic zones, such as Cafeteria Humabon, showed higher SFR and lower EE because more environmental changes triggered spike activity. This built-in responsiveness allows for automatic energy distribution across diverse building regions based solely on observed activity levels [22,23]. This selective activation arises from the LIF neuron’s threshold-and-leak dynamics: the membrane potential must accumulate sufficient input current to overcome both the leakage decay (λ = 0.02) and the dynamic threshold (θ = μ + 1.5σ), ensuring that only environmentally significant events trigger computational processing.

### 5.2. Neuron Scaling and Practical Deployability

The neuron scaling data in Table 4 expose a key insight into hardware setup: performance plateaus at four neurons per monitored parameter, maintaining stable SFR at 11. 07%, MRT at 11. 16 steps, and EE at 9. 03 regardless of population size, whether four, eight, or sixteen neurons are active. Saturation arises because the logical OR aggregation mechanism across the neuron population captures all threshold crossings even when individual responses are slightly delayed or jittered by hardware variability (σ_th_ = 0.05, σ_leak_ = 0.005). Once the population is large enough for at least one neuron to respond to every threshold crossing within one integration window, additional neurons contribute no new detections. As a result, the framework achieves complete event detection with just twenty-eight neurons total (four per seven IAQ parameters), representing minimal physical infrastructure demands. This outcome supports scalability claims advanced by Park et al. [22] and Xue et al. [17], suggesting efficient deployment on edge-level neuromorphic platforms without large resource expansion.

### 5.3. Robustness and Tunability

Performance remains consistent across multiple parameter settings in Table 5, illustrating resilience against configuration shifts rather than abrupt failures under stress. When thresholds are set at τ = 15/20 and V_th_ = 0.9/1.0, outputs remain identical, an indication of operational stability within a defined functional range. Configuration three introduces a higher brink (V_th_ = 1.1), leading to reduced firing rates (SFR drops to 7.38%), slower reaction times (MRT rises to 16.33 steps), and improved energy conservation (EE reaches 13.54). These changes reflect a measurable trade-off between sensitivity and efficiency, a feature useful for adjusting deployment strategies as context: elevated thresholds can be applied in power-limited devices, while lower thresholds support faster responses in safety-sensitive environments requiring immediate alerts [15]. This deterministic relationship between LIF parameters and output metrics follows directly from the integrate-and-fire dynamics: a higher V_th_ requires more input current accumulation before spike generation, reducing the probability and frequency of firing events while increasing the inter-spike interval.

### 5.4. Adaptive STDP Learning

Figure 3 illustrates how synaptic weights adjust automatically across zones without reliance on labeled data or explicit training cycles. In cafeteria settings, oscillatory potentiation and depression patterns emerge in response to recurring occupancy-fluctuation behavior consistent with temporal coding principles [18]. Stable paths observed in quiet library spaces show suppression of irrelevant signal spikes under minimal environmental variation [28]. This self-calibrating behavior is valuable in long-term sensor networks affected by seasonal shifts or changes in user behavior over time.

### 5.5. Validation of Cross-Paradigm Efficiency Metrics

The proposed PEI and CES metrics were formulated to resolve a notable absence in the existing literature—the lack of shared standards for evaluating event-driven vs. continuous processing setups operating under distinct computational goals [15]. The axial analysis in Section 3.6 sets up that CES fulfills necessary criteria: non-negativity, monotonicity, dimensional coherence, proper boundary limits, and compatibility across models—offering solid theoretical grounding for its use as an aggregate measure. Empirical testing through sensitivity analysis (Table 8) reveals that CES rankings remain stable even when the PEI-TE weightings vary a lot. Spearman rank correlations surpass 0.96 across all configurations tested due to clear separability among core indicators, cafeteria zones consistently display high sparsity combined with low energy costs even as auditoriums, and libraries reflect low spike rates paired with high consumption patterns, which generates reliable ordering regardless of minor parameter adjustments. Under the revised PEI/CES formulation, the neuromorphic LIF model achieved a PEI of 12.10 ± 3.39 and a CES of 0.445 ± 0.040. These results indicate strong performance per unit computational activity, although the composite efficiency score remained lower than the strongest continuous baselines due to the temporal-efficiency component. The stabilization effect observed is also attributable to AKF preprocessing, reducing mean noise by 5.11%, effectively minimizing false activations and improving consistency in downstream metrics like SFR and MRT across different zones [24].

### 5.6. Comparative Context with Literature

While a direct comparison with existing neuromorphic IAQ systems is not possible due to the absence of prior work in this specific domain, the results can be contextualized against related neuromorphic sensing applications. Lu and Xiao [19] reported 47% energy reductions in agricultural greenhouse SNN systems; the present framework’s 8.9-fold FLOP reduction (approximately 89% fewer operations) extends this principle to multi-parameter IAQ monitoring. Park et al. [22] demonstrated SNN-to-hardware mapping for edge-AI sensors without reporting per-sample FLOP counts, making a direct numerical comparison infeasible. The expanded baseline comparison shows that XGBoost (93.60%) and Logistic Regression (91.58%) outperform the neuromorphic model (87.88%) in classification accuracy, confirming that the neuromorphic approach’s advantage lies in computational efficiency rather than accuracy superiority. The 8.9-fold computational reduction must be weighed against the 5–6 percentage point accuracy gap when selecting the appropriate paradigm for a given deployment scenario. Table 9 presents a multi-criteria comparison using the eight evaluation dimensions specified by the reviewer, demonstrating that the proposed framework is the only work reporting quantified results across all criteria.

The comparison shows that the proposed framework is the only work among the reviewed studies that reports quantified results across all eight criteria. Existing neuromorphic sensing and edge-SNN studies generally report limited dimensions, such as energy reduction, toolchain mapping, or partial robustness, but do not jointly quantify sparse firing behavior, computational reduction, neuron scaling efficiency, energy proportionality, robustness, learning adaptivity, composite performance metrics, and preprocessing impact. In contrast, this study reports a mean SFR of 10.94%, an 8.9-fold computational reduction relative to LSTM inference, saturation at 4 neurons per IAQ parameter, strong energy proportionality indicated by an inverse SFR–EE correlation of r = −0.98, stable performance across LIF parameter configurations, adaptive STDP behavior, validated composite metrics, and AKF-based noise reduction. This supports the claim that the proposed framework provides a more comprehensive evaluation of neuromorphic IAQ monitoring than the selected related works.

### 5.7. Limitations

There are several limitations that have to be plainly stated. One of the limitations of this work is that the energy efficiency (EE) and the 8.9-fold computational reduction study were based on spike density and FLOP counts instead of actual power measurements of the hardware. To really get absolute energy savings in real working conditions, it is required to do a validation on dedicated neuromorphic processors (e.g., Intel Loihi, SpiNNaker, or FPGA-based SNN accelerators) [15,22].

Another limitation could be the cross-paradigm comparison between neuromorphic SFR and conventional model accuracy (Table 5 and Table 6) that in fact measure totally different things: SFR is a sparsity descriptor that accounts for event selectivity, whereas accuracy is a metric of the model’s prediction performance. Even though PEI and CES make a normalization of these within their paradigms, the resulting numerical values of the two paradigms cannot be compared directly. The best experimental setting would be to evaluate all the paradigms on a shared downstream task with common evaluation criteria, e.g., anomaly detection F1-score or pollutant threshold exceedance classification. For now, this is a very challenging issue, and therefore it is an important line of work for the future.

Third, while expanded baselines (GRU, Temporal CNN, XGBoost, and Logistic Regression) were added in the multi-seed validation, no exhaustive hyperparameter optimization was performed for any model. More sophisticated architectures or tuned hyperparameters could narrow or close the accuracy gap with the neuromorphic approach. Fourth, the data were collected from only one building complex for a limited period; generalization to other building types requires further validation. How the methods discussed in this paper will perform in other types of buildings—commercial, industrial, or residential—with different pollutant patterns, occupancy changes, and HVAC setups, as well as seasonal variations is still unknown. So, the results here, at best, are of single-site validation or a proof of concept.

Fifth, multi-seed validation (five seeds: 7, 21, 42, 84, and 168) was conducted across all nine rooms and seven models (Section 4.7). However, formal bootstrap uncertainty estimates and confidence intervals for SFR, MRT, and CES were not computed and should be addressed in future work.

Sixth, the sensor calibration was done according to the factory defaults, and no field recalibration, cross-sensitivity correction, or drift compensation was carried out. For long-term deployment, a periodical recalibration protocol is necessary to maintain spike encoding fidelity. Seventh, in the current framework, there is no definition of ground truth event labels that is independent of the spike encoding threshold. The dynamic threshold (θ = μ + 1.5σ) not only determines spike generation but also implicit event classification, thus establishing a circular dependency. External ground truths, such as expert-annotated IAQ events, regulatory threshold exceedances, or independent reference instruments, would allow for a more rigorous validation of event detection accuracy.

## 6. Conclusions

This article introduces a spike-driven neuromorphic processing framework as a solution for energy-efficient IAQ monitoring in multi-zone buildings. The framework combines AKF data preprocessing (mean noise reduction: 5.11%), a dynamic threshold spike encoder, and LIF-STDP neuron models. Results from nine zones with seven IAQ parameters showed: an average SFR of 10.94%, MRT of 10.62 timesteps, EE of 9.28, and approximately 89% less computational cost (a factor of 8.9 reduction) than continuous baselines. Neuron scaling experiments indicated that four neurons per parameter were sufficient to achieve good performance, while robustness testing showed stable operation with various LIF parameters.

Three aspects of this paper move the research frontier ahead: (1) a neuromorphic framework for multi-parameter, multi-zone IAQ monitoring with neuron scaling and robustness characterization; (2) an AKF-to-spike encoding pipeline for raw environmental sensors to neuromorphic computation; and (3) revised PEI/CES metrics for principled cross-paradigm evaluation, also validated by axiomatic analysis and sensitivity testing (Spearman ρ > 0.96 for all weighting configurations). The CES showed consistently energy-proportional performance (mean = 0.445, SD = 0.040) in all environments. This establishes spike-driven neuromorphic computation as a scalable basis for decentralized, low-power IAQ control in smart building sensor networks of the future.

The applicable boundaries of these conclusions are as follows: the framework has been validated only in a single educational building environment in a tropical climate, and the 8.9-fold computational reduction is a FLOP-based estimate (approximately 89% fewer operations). Future research should address: (1) hardware validation on dedicated neuromorphic processors (Intel Loihi and SpiNNaker) for absolute power savings; (2) multi-building deployment across diverse building types and climates; (3) integration of independent ground truth event labels for rigorous event detection evaluation; (4) closed-loop ventilation control demonstrations; and (5) long-term drift compensation and recalibration protocols for sustained field deployment.

## Figures and Tables

**Table 1:** Descriptive statistics of the indoor air quality dataset.

Parameter	Unit	Sample Count (n)	Minimum	Maximum	Range	Mean	Standard Deviation (SD)	Mean ± SD
PM_1_	µg/m^3^	524,040	0.4	378	0.40–378.00	8.09	8.74	8.09 ± 8.74
PM_2.5_	µg/m^3^	524,040	0.4	404	0.40–404.00	9.12	9.58	9.12 ± 9.58
PM_10_	µg/m^3^	524,040	0.5	474	0.50–474.00	9.9	10.87	9.90 ± 10.87
CO_2_	ppm	606,997	400	9999	400.00–9999.00	679.43	526.62	679.43 ± 526.62
CO	ppm	559,510	7	1000	7.00–1000.00	329.13	303.67	329.13 ± 303.67
TVOCs	ppb	606,997	0	59,400	0.00–59,400.00	589.16	7070.88	589.16 ± 7070.88
O_3_	ppb	476,553	0.06	6.56	0.06–6.56	1.23	1.12	1.23 ± 1.12
Temperature	°C	606,997	13.8	37.2	13.80–37.20	30.50	2.72	30.50 ± 2.72
Relative Humidity	%	606,997	0	100	0.00–100.00	59.56	8.06	59.56 ± 8.06

**Table 2:** Noise variance reduction by adaptive Kalman filter (AKF) preprocessing.

Monitored Zone	Noise Reduction (%)
Auditorium Right	2.62
809 A Classroom	19.37
Cafeteria Humabon	4.29
Cafeteria SLEX	8.43
Library Circulation SLEX	1.68
Library Lounge	2.80
Library Open Humabon	3.10
Library Open SLEX	2.40
Multipurpose Hall 1 (MPH1) Humabon Side	1.32
Overall Mean	5.11

**Table 3:** Neuromorphic model performance metrics across all monitored building zones.

Monitored Zone	SFR (%)	MRT (Steps)	EE (1/Spike Density)
Cafeteria SLEX	12.71	10.12	7.87
Cafeteria Humabon	12.92	9.83	7.74
Library Circ. SLEX	11.55	8.99	8.66
Library Lounge	10.95	10.05	9.13
Library Open Humabon	11.18	10.43	8.94
Library Open SLEX	10.75	11.38	9.30
Multipurpose Hall 1 (MPH1) Humabon Side	10.10	11.99	9.90
Auditorium Right	9.72	10.37	10.29
Classroom 809-A	8.53	12.44	11.72
Overall Mean	10.94	10.62	9.28

**Table 4:** Effect of neuron population size on neuromorphic model performance metrics.

Neurons Per Parameter	SFR (%)	MRT (Steps)	EE
1	5.52	22.23	18.11
2	7.09	17.36	14.10
4	11.07	11.16	9.03
8	11.07	11.16	9.03
16	11.07	11.16	9.03

**Table 5:** Robustness of neuromorphic model under different LIF parameter configurations (8 neurons per parameter).

τ	V_th_	λ	SFR (%)	MRT (Steps)	EE
15	0.90	0.015	11.07	11.16	9.03
20	1.00	0.020	11.07	11.16	9.03
25	1.10	0.025	7.38	16.33	13.54

**Table 6:** Comparative operational metrics across monitored zones. SFR = Sparse Firing Ratio; MRT = Mean Response Time (steps); EE = Energy Efficiency.

Zone	SFR (%)	LSTM Acc. (%)	Reg. Acc. (%)	MRT	EE
Auditorium Right	9.72	93.70	93.70	10.37	10.29
809 A Classroom	8.53	79.24	79.52	12.44	11.72
Cafeteria Humabon	12.92	65.00	64.51	9.83	7.74
Cafeteria SLEX	12.71	62.45	65.12	10.12	7.87
Lib. Circ. SLEX	11.55	62.98	62.10	8.99	8.66
Library Lounge	10.95	66.35	66.16	10.05	9.13
Lib. Open Humabon	11.18	47.35	49.68	10.43	8.94
Lib. Open SLEX	10.75	62.32	64.64	11.38	9.30
MPH1 Hum. Side	10.10	67.55	68.96	11.99	9.90
Overall Mean	10.94	67.44	68.27	10.62	9.28

**Table 7:** Revised Performance Efficiency Index (PEI) and composite efficiency score (CES) across evaluated models.

Model	PEI	CES	Interpretation
Neuromorphic LIF	12.10 ± 3.39	0.445 ± 0.040	Highest performance per unit computational activity; lower CES due to event-response timing penalty
XGBoost	0.94	0.648	Highest composite efficiency among continuous baselines
Logistic Regression	0.92	0.634	Strong accuracy-efficiency balance
Linear Regression	0.86	0.586	Stable baseline performance
GRU	0.85	0.579	Moderate deep-learning baseline performance
LSTM	0.81	0.551	Lower efficiency under the tested configuration
Temporal CNN	0.76	0.515	Lowest composite score among evaluated baselines

**Table 8:** Spearman rank correlation (ρ) between baseline neuromorphic CES zone ranking (α = 0.5) and rankings at alternative α values.

α Value	Emphasis Shift	ρ (Spearman)	Ranking Changes
0.30	TE-dominant	0.983	1 adjacent swap
0.40	Slight TE emphasis	0.998	0
0.50	Baseline (equal)	1.000	—
0.60	Slight PEI emphasis	0.998	0
0.70	PEI-dominant	0.967	2 adjacent swaps

**Table 9:** Multi-criteria comparison of the proposed framework with related neuromorphic sensing works.

Study/Work	Application/Focus	SFR	CR	NSE	EP	ROB	LA	CM	PI
Lu & Xiao [19]	Agricultural SNN	Not reported	47% energy reduction	N/A	Partial	Not tested	N/A	N/A	N/A
Park et al. [22]	Edge-AI SNN mapping	Not reported	Not reported; FLOP counts unavailable	N/A	N/A	Not tested	N/A	N/A	N/A
Xue et al. [17]	EdgeMap toolchain	Not reported	Toolchain-level only; not application level	N/A	N/A	N/A	N/A	N/A	N/A
Morshed et al. [18]	Stochastic SNN	Not reported	Not reported	N/A	N/A	Partial; stochastic vs. deterministic comparison	N/A	N/A	N/A
This study	Spike-driven neuromorphic IAQ monitoring	10.94% mean across 9 zones	8.9-fold vs. LSTM; 0.28 vs. 2.50 kFLOPs/sample	4 neurons/parameter; saturation point	Yes; inverse SFR–EE correlation, r = −0.98	Stable across 3 LIF configurations; CV < 5%	Yes; STDP potentiation/depression cycles adapt to zone-specific pollutant dynamics	PEI = 12.10 ± 3.39; CES = 0.445 ± 0.040; Spearman ρ > 0.96	AKF mean noise reduction = 5.11%

## Data Availability

The data used in this study are not publicly available due to institutional restrictions and data management policies but are available from the corresponding author upon reasonable request. A sanitized sample dataset, along with the full codebase for data preprocessing, feature selection, model training, and interpretability analysis, can be shared for academic and non-commercial use.

## Abbreviations

The following abbreviations are used in this manuscript:

AKF	Adaptive Kalman Filter
CES	Composite Efficiency Score
CO	Carbon Monoxide
CO_2_	Carbon Dioxide
CV	Coefficient of Variation
EE	Energy Efficiency
FLOPs	Floating-Point Operations
GPU	Graphics Processing Unit
IAQ	Indoor Air Quality
IoT	Internet of Things
kFLOPs	Thousand Floating-Point Operations
LIF	Leaky Integrate-and-Fire
MOS	Metal-Oxide Semiconductor
MRT	Mean Response Time
O_3_	Ozone
PEI	Performance Efficiency Index
PM	Particulate Matter
PM_1_	Particulate Matter ≤ 1 µm
PM_2.5_	Particulate Matter ≤ 2.5 µm
PM_10_	Particulate Matter ≤ 10 µm
SD	Standard Deviation
SFR	Sparse Firing Ratio
SNN	Spiking Neural Network
STDP	Spike-Timing-Dependent Plasticity
TE	Temporal Efficiency
TVOCs	Total Volatile Organic Compounds
